# Adjuvant Chemotherapy for Brain Tumors Delivered via a Novel Intra-Cavity Moldable Polymer Matrix

**DOI:** 10.1371/journal.pone.0077435

**Published:** 2013-10-14

**Authors:** Cheryl V. Rahman, Stuart J. Smith, Paul S. Morgan, Keith A. Langmack, Phil A. Clarke, Alison A. Ritchie, Donald C. Macarthur, Felicity R. Rose, Kevin M. Shakesheff, Richard G. Grundy, Ruman Rahman

**Affiliations:** 1 Division of Drug Delivery and Tissue Engineering, School of Pharmacy, University of Nottingham, Nottingham, United Kingdom; 2 Children’s Brain Tumor Research Centre, School of Clinical Sciences, University of Nottingham, Nottingham, United Kingdom; 3 Division of Pre-Clinical Oncology, University of Nottingham, Nottingham, United Kingdom; 4 Medical Physics, Nottingham University Hospitals, Nottingham, United Kingdom; 5 Department of Neurosurgery, University Hospital, Queen’s Medical Centre, Nottingham, United Kingdom; Wake Forest University, School of Medicine, United States of America

## Abstract

**Introduction:**

Polymer-based delivery systems offer innovative intra-cavity administration of drugs, with the potential to better target micro-deposits of cancer cells in brain parenchyma beyond the resected cavity. Here we evaluate clinical utility, toxicity and sustained drug release capability of a novel formulation of poly(lactic-co-glycolic acid) (PLGA)/poly(ethylene glycol) (PEG) microparticles.

**Methods:**

PLGA/PEG microparticle-based matrices were molded around an *ex* *vivo* brain pseudo-resection cavity and analyzed using magnetic resonance imaging and computerized tomography. *In* *vitro* toxicity of the polymer was assessed using tumor and endothelial cells and drug release from trichostatin A-, etoposide- and methotrexate-loaded matrices was determined. To verify activity of released agents, tumor cells were seeded onto drug-loaded matrices and viability assessed.

**Results:**

PLGA/PEG matrices can be molded around a pseudo-resection cavity wall with no polymer-related artifact on clinical scans. The polymer withstands fractionated radiotherapy, with no disruption of microparticle structure. No toxicity was evident when tumor or endothelial cells were grown on control matrices *in* *vitro*. Trichostatin A, etoposide and methotrexate were released from the matrices over a 3-4 week period *in* *vitro* and etoposide released over 3 days *in* *vivo*, with released agents retaining cytotoxic capabilities. PLGA/PEG microparticle-based matrices molded around a resection cavity wall are distinguishable in clinical scanning modalities. Matrices are non-toxic *in* *vitro* suggesting good biocompatibility *in* *vivo*. Active trichostatin A, etoposide and methotrexate can be incorporated and released gradually from matrices, with radiotherapy unlikely to interfere with release.

**Conclusion:**

The PLGA/PEG delivery system offers an innovative intra-cavity approach to administer chemotherapeutics for improved local control of malignant brain tumors.

## Introduction

Central nervous system (CNS) tumors are the major cause of cancer related death in children and adults up to the age of 40. This is particularly the case for children with high grade glioma (HGG), incompletely resected ependymoma (especially those under 3 years of age) and primitive neuroectodermal tumors (PNET) who have frequent tumor recurrence and poor survival rates despite multimodal therapy [1–3]. Despite the invasive nature of high grade glioma, most relapses occur within the wall of the surgical resection cavity, as do most ependymoma relapses. A subset of HGG (potentially identifiable on advanced MRI) may display limited invasion and be particularly amenable to effective localized therapy [4]. In adults, glioblastoma multiforme (GBM) is the most frequent and aggressive primary brain tumor and despite radical surgery, radiotherapy and chemotherapy, median survival after diagnosis remains only 14 months [5]. Conventional oral or intravenous chemotherapy distributes drugs to the whole body whereby toxicity of the drug limits the maximum dose achievable within the targeted tumor site, particularly for CNS tumors where the blood brain barrier (BBB) restricts drug influx from the circulation. There is therefore a need to develop more effective and targeted chemotherapy regimes that can eradicate residual brain tumor cells following surgical resection, thereby improving local control by targeting neoplastic cells within brain parenchyma beyond the surgical cavity wall and reducing the risk of tumor recurrence [6]. 

Undesired toxicity as a result of systemic administration of chemotherapeutics and drug doses below the therapeutic window at the tumor site (due to the BBB) are the driving force behind the development of local drug delivery systems. Furthermore, most recurrences of high grade tumors occur within ~2cm of the wall of the resection cavity. The opportunity to deliver therapeutic cancer drug concentrations locally creates the possibility of improving both the safety (low toxic dose systemically) and efficacy (high effective dose locally) of cancer chemotherapy, thereby enhancing the benefit of surgery, as well as continuing anti-neoplastic treatment during the interval between surgery and commencement of systemic adjuvant therapy. 

Drug delivery technologies have developed from simple tablets, liquids and injectables to sophisticated systems utilizing bioengineered products. Many of these emerging products use polymer-based drug delivery systems including microspheres and nanoparticles, each technology offering unique characteristics to optimize drug delivery [7]. Such drug delivery modes may be an important armament for the future treatment of malignant brain tumors as they encompass strategies to enhance BBB penetration, to specifically target brain tumor cells and to implant the polymer at close proximity to residual cancer cells [8–16]. For example, OncoGel™, a controlled-release formulation of paclitaxel in ReGel™ has shown much pre-clinical promise. This system comprises a thermosensitive triblock copolymer (PLGA-PEG-PLGA) which is water soluble at 2-15°C and turns into a viscous gel at body temperature [8,17]. Pre-clinical and early clinical investigations demonstrated OncoGel™’s ability to physically target paclitaxel to esophageal and brain tumor tissue via intralesional injection into the tumor cavity following resection, with an acceptable safety profile and moderate increase in survival in a rat gliosarcoma model [16,18,19]. The rationale of these polymer-based approaches is to improve upon drug efficacy, increase exposure time of tumor cells to drug, protect drugs from degradation and clearance by the immune system until its release from the polymer, reduce the debilitating sequelae of current systemic chemotherapeutics and to allow oncological treatment to be maintained in the interval between surgery and radiotherapy [20,21]. 

Although a myriad of drug-polymer devices have been developed to date, the Food and Drug Administration (FDA) and National Institute for Health and Clinical Excellence (NICE) has solely approved the use of chemotherapy impregnated polymeric wafers (Gliadel®) for local chemotherapy delivered via a biomaterial, for the treatment of primary and recurrent malignant glioma. These wafers which are neurosurgically implanted at the time of tumor resection, gradually release the chemotherapeutic agent carmustine, which then diffuses into the surrounding brain and targets the residual cancer cells that have infiltrated the brain tissue. These studies and trials offer hope to this mode of intra-cavity drug delivery, with results showing a moderate but significant survival benefit of 2.3 months and 1.8 months median survival for newly diagnosed and recurrent high grade gliomas respectively [22–24]. Nevertheless the treatment has shown limited efficacy mainly due to: (i) poor drug diffusion, restricted to 2-3mm bordering the implant; (ii) implants not maintaining close contact with the resection cavity rim and potentially falling to the bottom of the cavity; (iii) only one drug being delivered [25]. In the case of Gliadel® the biomaterial was specifically engineered around the drug carmustine, rather than offer a drug delivery system widely applicable for the delivery of other chemotherapeutic agents.

Shakesheff and colleagues previously described a novel temperature-sensitive and biodegradable formulation based on blended poly(lactic-co-glycolic acid) (PLGA) and poly(ethylene glycol) (PEG) microparticles [26–28]. This technology has successfully been used to deliver an oesteogenic growth factor for bone repair in a murine calvarial defect model [29]. The microparticles are a free-flowing powder at room temperature and create a paste when mixed with a saline-based carrier solution. The formulation can be injected or pasted at room temperature and then gradually solidifies (sinters) into a solid, porous matrix at body temperature. Multiple chemotherapeutic agents can be loaded into the carrier solution phase that is mixed with the microparticles to form a paste. The chemotherapeutic agents adsorb to the surface of the microparticles and absorb into the microparticle bulk structure. This paste can then be molded around the resection cavity wall. Within approximately 15 minutes at body temperature, the particles solidify into a porous matrix which then gradually releases the chemotherapy. Solidification only at body temperature (a process which is not exothermic) aids retention of the drug-loaded matrix within the cavity wall. PLGA polymer degrades via hydrolysis over a period of months and is removed from the body through excretion of the lactic and glycolic acid by-products via metabolic pathways such as the tricarboxylic acid cycle [30]. PLGA was chosen because it has a long history of use as a FDA-approved degradable medical implant material with well established safety in the clinic (for example low cytotoxicity and good biocompatibility of resorbable sutures) [31–33]. Importantly, PLGA microspheres gradually degrade and have good biocompatibility to brain tissue [34,35]. The properties of the PLGA/PEG microparticle based-matrices potentially offer several advantages over systemic administration and current polymeric delivery systems: (i) matrices can be molded into any size or shape desired, such as the irregular surgical resection cavity wall; (ii) capability to incorporate and release multiple drugs increasing the flexibility of treatment offered to the clinician and patient; (iii) sustained drug release over 1-3 weeks providing oncological treatment in the interval before commencement of post-operative chemo/radiotherapy.

Here we demonstrate the novel mode of application of the PLGA/PEG microparticle-based matrix drug delivery system for brain tumors for which complete surgical resection is not achievable. We describe its clinical compatibility with respect to radiotherapy and magnetic resonance imaging/computerized tomography (MRI/CT) scanning modalities. Our laboratory-based studies evaluate *in vitro* sustained release of trichostatin A (TSA), etoposide (ETOP) and methotrexate (MTX) from PLGA/PEG microparticle-based matrices, short-term *in vivo* release of etoposide and assess whether released agents retain cytotoxic function.

## Materials and Methods

### PLGA/PEG particle production

Thermosensitive particles were fabricated from blends of 53kDa P_DL_LGA (85:15 DLG 4CA) (Lakeshore Biomaterials, USA) and PEG 400 (Sigma Aldrich, UK) as previously described [26]. Briefly, a mixture of 93.5%:6.5% PLGA:PEG (*w/v*) was blended at 80-90°C on a hotplate. The melted PLGA and PEG were mixed together by hand using a PTFE-coated spatula and allowed to cool. Polymer blend sheets were then ground into particles in a bench-top mill (Krups Mill F203) and the particles were sieved to obtain the 100-200µm particle size fraction. 

### Matrix preparation

Composite matrices were prepared in PTFE moulds. For *in vitro* release experiments cylindrical scaffolds of 12mm length and 6mm diameter were produced. The PLGA/PEG particles were mixed manually with drug solution (trichostatin A, etoposide or methotrexate) or water (negative controls). A ratio of 1:0.6 of particles to solution was used. The drug loaded matrices contained 50µg of drug per matrix. The particle paste was then packed into the mould which was placed at 37°C for 2 hours to allow matrix formation (sintering). For *in vitro* biocompatibility and toxicity assays cylindrical scaffolds 1mm thick and 4mm diameter were produced. The PLGA/PEG particles were mixed manually with drug solution (trichostatin A, etoposide or methotrexate) at a ratio of 1:0.6 of particles to solution. The drug loaded matrices contained 50µg of drug per matrix. The particle paste was then packed into the mould which was placed at 37°C for 2 hours to allow matrix formation.

### Experimental cell lines and culture

PFSK-1 (childhood central nervous system primitive neuroectodermal tumor) (CNS PNET)), DAOY (childhood medulloblastoma), U87 (adult glioblastoma multiforme) and C6 (rat glioma) cell lines were previously characterized by and purchased from ATCC. Monolayer cells were cultured in DMEM (Sigma, UK), supplemented with 10% fetal bovine serum (PAA Labs, UK), 5mM sodium pyruvate, 5mM L-Glutamine and maintained in a humidified incubator at 37°C and 5% CO_2_. To assess *in vitro* biocompatibility and toxicity of PLGA/PEG matrices cells were seeded in triplicate. Matrices were placed in single wells of a 24-well plate and pre-treated with 50µl of culture media. A 30µl suspension containing 1x10^5^ cells were placed onto each matrix and plates incubated for 2 hours at 37°C to allow cells to adhere to the polymeric microparticles. Fresh culture media (1ml) was added to each well ensuring matrices were submerged, and incubated at 37°C until required for assaying.

### *Ex vivo* application of PLGA/PEG matrices

Sheep heads were obtained from a local abattoir after permission was granted to use these animal parts (C Brumpton Butchers Ltd, Nottingham). Heads were fixed in a vice and bilateral skin and muscle flaps raised. Three burrholes were performed with a Hudson brace on each fronto-temporal region of the cranium and joined with a Gigli saw to raise craniotomies. The dura was incised and flapped back, and secured with vicryl stay sutures. Incisions were made through the pia and the brain parenchyma excised to give a cavity of dimensions 2.5x2.5x2.5cm. PLGA/PEG blended microparticles were mixed with PBS in a 1:0.6 ratio to form polymer-based paste and applied to the ovine pseudo-resection cavity. Cavities were either filled completely with PLGA/PEG or lined all around to an approximate depth of 2mm.

### MRI and CT scanning of *ex vivo* brain

MR imaging was performed using a clinical 3 T Achieva MR scanner (Philips Medical, Best, The Netherlands) with the specimen placed inside the 8 channel receive-only head coil. Routine fat-suppressed 3D turbo spin echo T1- and T2-weighted whole brain imaging was performed with acquisition resolution of 1x1x1 mm, 192x192x160 matrix, echo train length of 133, echo time of 262 ms, repetition time of 2500 ms, and 464 s overall scan time. The CT scans were acquired using an adult head protocol on a clinical Mx8000 IDT 16 scanner (Philips Medical, Best, The Netherlands). 144 slices, 1.5 mm thick, were acquired at 120 kV with 512x512 matrix and 0.45 mm in-pixel pixel size.

### Chemotherapeutic and experimental agents

TSA and ETOP were purchased from Sigma, UK and resuspended in dimethyl sulfoxide (DMSO) to give 5mM and 50mM working concentrations respectively. MTX was purchased from Sigma, UK and resuspended in phosphate buffered saline to give a 100mg/ml stock solution.

### Radiotherapy dosing regime

A 12mm x 6mm sample of the biomaterial was placed within a cavity of the head region of an anthropomorphic phantom (Rando^TM^ phantom, The Phantom Laboratory, Salem, NY, USA). The head area was then CT scanned to give a set of contiguous 3 mm slices (Aquilion LB CT scanner, Toshiba, Crawley, UK). These CTs were transferred to a radiotherapy treatment planning system (Oncentra version 3.3 SP3, Nucletron, UK) using a DICOM protocol. Within the planning system the sample material was outlined and a region of interest 1 cm in all directions was grown to provide a target volume. An experienced radiotherapy planner then devised a treatment plan to irradiate the target volume as uniformly as possible to 60 Gy using a 6 MV beam. A standard isocentric 3 field arrangement was used, which gave a mean target dose of 60 Gy (range 59.3 Gy to 60.7 Gy). The plan was delivered as 30 daily 2 Gy fractions using a clinical linear accelerator (Elekta Precise, Elekta, Crawley, UK). The fractionation schedule was as routine clinical practice, i.e. one fraction per day Monday to Friday, no irradiations at the weekends.

### Live/Dead viability assay

Brain tumor cells were seeded onto PLGA/PEG matrices as described previously and Live/Dead™ viability/cytotoxicity assay (Invitrogen, UK) conducted on days 1,2 and 3 post-seeding. 5µl of 2mM ethidium homodimer-1 and 2.5µl of 4mM calcein AM were added to 10ml DMEM and 1ml of the final solution was used to stain and completely submerge one cell-seeded PLGA/PEG matrix (one matrix per well of 24-well plate). Following incubation for one hour at 37°C, matrices were rinsed three times with DMEM to remove background staining and a further three times with PBS. Each matrix was carefully placed on a microscope slide and fluorescence signals (green = viable; red = dead) visualized using a Leica DMRB upright fluorescent microscope. Matrices were placed back into culture to ensure tracking of the cells present on the same matrix over 3 days with respect to viability observations and quantitation of fluorescent cells. Three matrices were used for each cell line and 100 cells were counted for each matrix to determine the proportion of live:dead cells.

### Alamar Blue proliferation assay

Brain tumor cells were seeded onto PLGA/PEG matrices in 24-well plates as described above. On days 1, 2 and 3 post-seeding, matrices were washed three times with warm PBS and finally placed in 1ml PBS. 100µl Alamar Blue indicator dye (Invitrogen, UK) was added to each well ensuring matrices were submerged. Following incubation for 90 minutes at 37°C, 100µl aliquots were transferred to single wells of a 96-well black-bottom plate in triplicate and fluorescence emission measured at 585nm using a plate reader (Tecan, Switzerland). Three matrices were used for each cell line and fluorescent readings were measured for each matrix in triplicate. Matrices were placed back into a single well of a 24-well plate and fresh culture media added to ensure tracking of the same matrix over 3 days with respect to cell proliferation. Fluorescence intensity over a 3-day culture period was compared to counterpart monolayer cells. Data is presented as the average relative fluorescence units of three independent experiments with error bars indicating standard error of the mean values.

### Scanning electron microscopy

Cell-seeded PLGA/PEG matrices were washed three times in fresh PBS and fixed in 3% glutaraldehyde for 12 hours. Fixed samples were then washed three times in PBS and dehydrated for a further 12 hours by exposure to air at room temperature. Fixed and dehydrated cell-seeded matrices were mounted on aluminium stubs and sputter-coated with gold at an argon current rate of 30mA for 3 minutes. Cell attachment and morphology on the matrices was visualized using a scanning electron microscope (SEM) (JEOL JSM-6060LV) at 10 kV. 

### *In vitro* drug release from PLGA/PEG matrices

50µg or 100µg drug-loaded matrices were placed in 4ml fresh PBS buffer pH 7.4 and incubated at 37°C. At various time intervals, the entire volume of PBS was replaced with 4ml fresh release buffer and 100µl of buffer containing released drug was sampled in triplicate at a relevant UV wavelength (TSA, 342nm, ETOP, 295nm and MTX, 324nm) and concentration of drug measured using a standard curve determined at the same time. Three matrices were used for each drug and blank PBS loaded PLGA/PEG matrices were used as a control for background absorbance values. Assays were terminated when 80% or more of the drug had been released and when no drug release was detected for two days or more. Data is presented as cumulative drug release as a function of time.

### Cytotoxicity of released agents

50µg drug-loaded matrices were placed in 2ml of culture media in single wells of a 24-well plate. After either 24 hours or 14 days, media was removed and brain tumor cells were seeded onto matrices using a 30µl suspension containing 1x10^5^ cells. Cells were allowed to adhere onto matrices for two hours prior to adding 2ml fresh culture media to the wells. Alamar Blue proliferation assay (Invitrogen, UK) was conducted 72 hours post-cell seeding and percentage viability of cells relative to untreated cells was measured. Data is presented as the average relative fluorescence units of three independent experiments with error bars indicating standard error of the mean values.

### *In vivo* etoposide release from PLGA/PEG matrices

This study was approved by the University of Nottingham local Ethical Review Committee and granted by the UK Home Office (License No. PPL 40/3559), after consideration of the justification of animal research and good animal welfare. Six 4-6 week old male MF-1 nude mice (3 mice per arm) were maintained under standard conditions as detailed in the UK Home Office Animals (Scientific Procedures) Act 1986 and studies conducted and reported in compliance with the 2010 NC3R ARRIVE guidelines. Animals U87 GBM cells tagged with a bioluminescent marker (DLuX) were injected subcutaneously into the left flank and the tumour grown for 15 days whilst monitoring using the IVIS Spectrum bioluminescent imaging system (PerkinElmer, UK). Mice with satisfactory tumour take and growth rates underwent partial tumour resection. The previous flank incision was re-opened and a biopsy punch/fine suction tip used to resect tumour back to the tumour/tissue interface, thus mimicking the surgical technique utilized in human patients undergoing comparable surgery for GBM. Etoposide-loaded PLGA/PEG matrices (experimental arm) or blank PLGA/PEG matrices (control arm) were moulded around the resection cavity. Animals were weighed daily by an experienced technician, any adverse effects noted, and sacrificed using cervical dislocation once their clinical condition deteriorated, in order to ameliorate suffering. Mice were sacrificed 3 days post-implantation and tumour tissue sectioned prior to staining with hematoxylin and eosin or glial fibrillary acidic protein (GFAP) (anti-GFAP (Abcam, ab726; 1:1000).

### Statistics

Pearsons correlation coefficient was used to determine the concordance of drug release rates between PLGA/PEG formulations with two different drug concentrations. Unpaired student t-test was used to determine whether proliferation rates of cells cultured on matrices differed significantly from corresponding cells cultured as monolayers with a p-value < 0.05 deemed statistically significant. Statistical analyses were conducted using SPSS software version 6.

## Results

### PLGA/PEG microparticle-based matrix can be molded around a resection cavity wall

**Figure 1:**
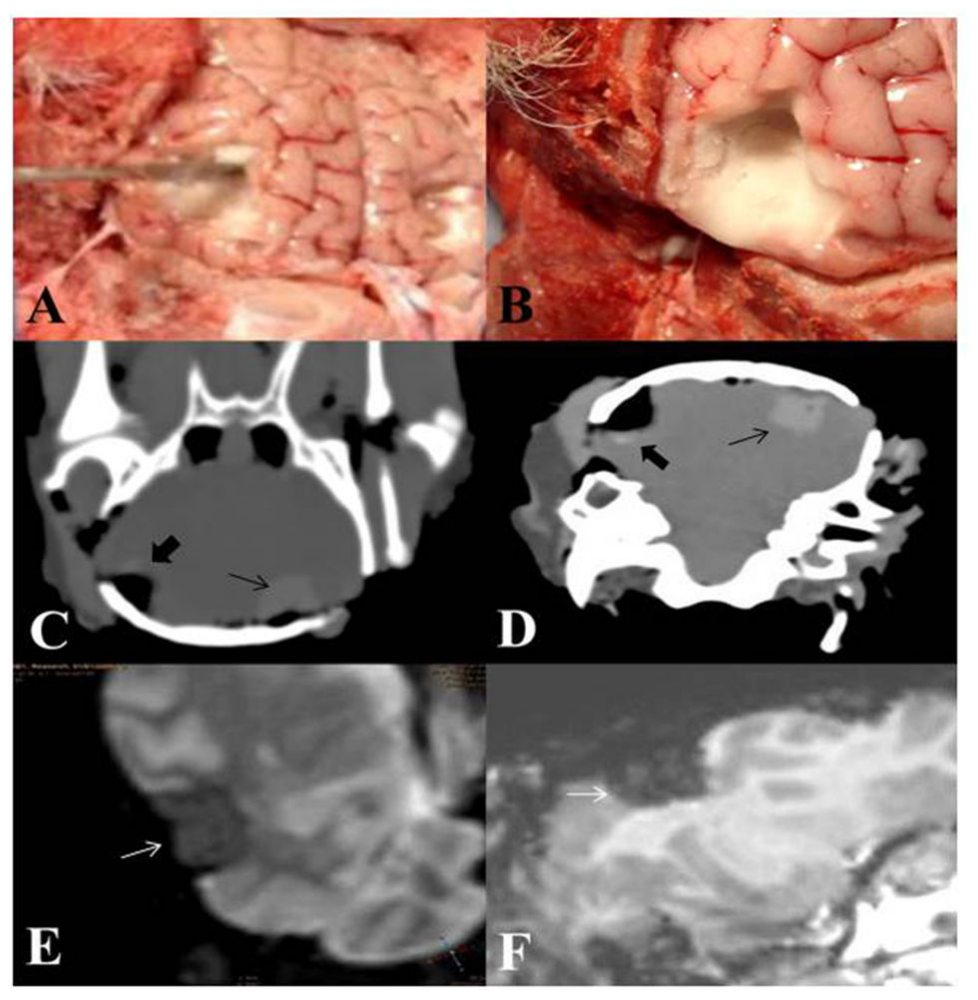
Application of PLGA/PEG microparticle-based matrices to a surgical resection cavity *ex* *vivo*. (A) When mixed with a saline carrier solution, PLGA/PEG microparticles form a paste that can be molded around an ovine pseudo-resection cavity *ex* *vivo*. (B) Within 15 minutes at 37°C, particles solidify to form a matrix that retains close apposition to the resection cavity wall. (C-D) Computerized tomography scans of *ex* *vivo* ovine brain demonstrating clearly distinguishable pseudo-resection cavity filled with PLGA/PEG (black arrows) and second cavity lined with PLGA/PEG (black arrowheads). (E-F) T2 and T1 weighted magnetic resonance imaging scan of *ex* *vivo* ovine brain with clearly distinguishable pseudo-resection cavity filled with PLGA/PEG (white arrows).

The PLGA/PEG paste was easily applied to and readily molded around, an irregular-shaped pseudo-resection cavity wall *ex vivo* (Figure 1a and Movie S1). The polymer formulation fully maintained its shape after 15 minutes sintering at 37°C, with close apposition to the cavity wall (Figure 1b and Movie S2).

### PLGA/PEG microparticle-based matrix can be distinguished by MRI and CT scanning

To establish whether PLGA/PEG interferes with MRI- and CT-based brain scans by causing image artifacts that obscure visualization of brain parenchyma and thereby potentially hampering identification of a recurrent tumor, an ovine head containing one polymer-filled and one polymer-lined pseudo-resection cavity was scanned *ex vivo* under standard clinical procedure. Cavities filled and lined with PLGA/PEG are distinguishable from the surrounding brain parenchyma using standard CT scanning (Figure 1 c-d). Similarly, cavities filled with PLGA/PEG are distinguishable from surrounding brain parenchyma using T2- and T1- weighted MRI scans (Figure 1 e-f respectively). Edges of the cavity are clearly defined with no additional image artifacts observed from the PLGA/PEG, indicating insignificant spatial distortion and good contrast with brain parenchyma on T2 and T1 MR (Figure 1c). Therefore application of PLGA/PEG matrices does not interfere with clinical scanning modalities used as standard procedures for the detection of brain tumors.

### Radiotherapy does not alter PLGA/PEG matrix microstructure

**Figure 2:**
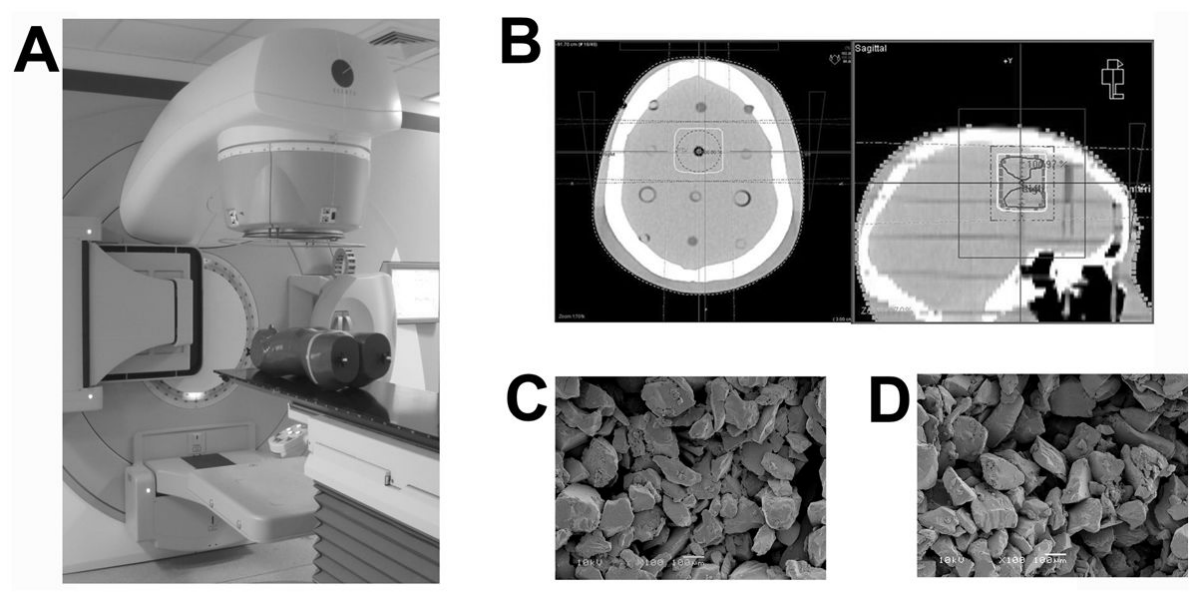
Effect of clinically-relevant radiotherapy regime on PLGA/PEG microparticle-based matrices. (A) PLGA/PEG matrices (12mm x 6mm) were placed within cavities in the brain area of an anthropomorphic phantom. (B) A head computerized tomography was obtained (3mm slices) using a standard radiotherapy protocol and the computerized tomography transferred to a radiotherapy planning system to give a uniform standard isocentric three field 60 Gy dose (30 daily 2 Gy fractions) of radiation to the biomaterial and surrounding area. (C) Scanning electron microscopy of control non-irradiated matrices kept at room temperature for 30 days, showing distinct and structurally intact microparticles with visible pores between particles. (D) Scanning electron microscopy of irradiated matrices showing no obvious difference in matrix microstructure (scale bar 100µm).

In anticipation of radiotherapy treatment for patients with PLGA/PEG intra-cavity implants, it is important to determine effects of radiation on the biomaterial and subsequent drug release. Moreover the effect of radiation on the microstructure of the PLGA/PEG matrix is unknown. PLGA/PEG matrices, placed in the brain area of an anthropomorphic phantom were subjected to a standard isocentric high dose radiotherapy regime of 60 Gy (2 Gy fractions daily for 30 days) (Figures 2a-b). Irradiated matrices show no visible difference in terms of microparticle size, morphology and distribution throughout the matrices when compared to control non-irradiated matrices stored under similar conditions. Microparticles appear structurally intact with visible pores between microparticles in both cases (Figure 2c-d).

### PLGA/PEG matrices are non-toxic to normal and cancerous cells

**Figure 3:**
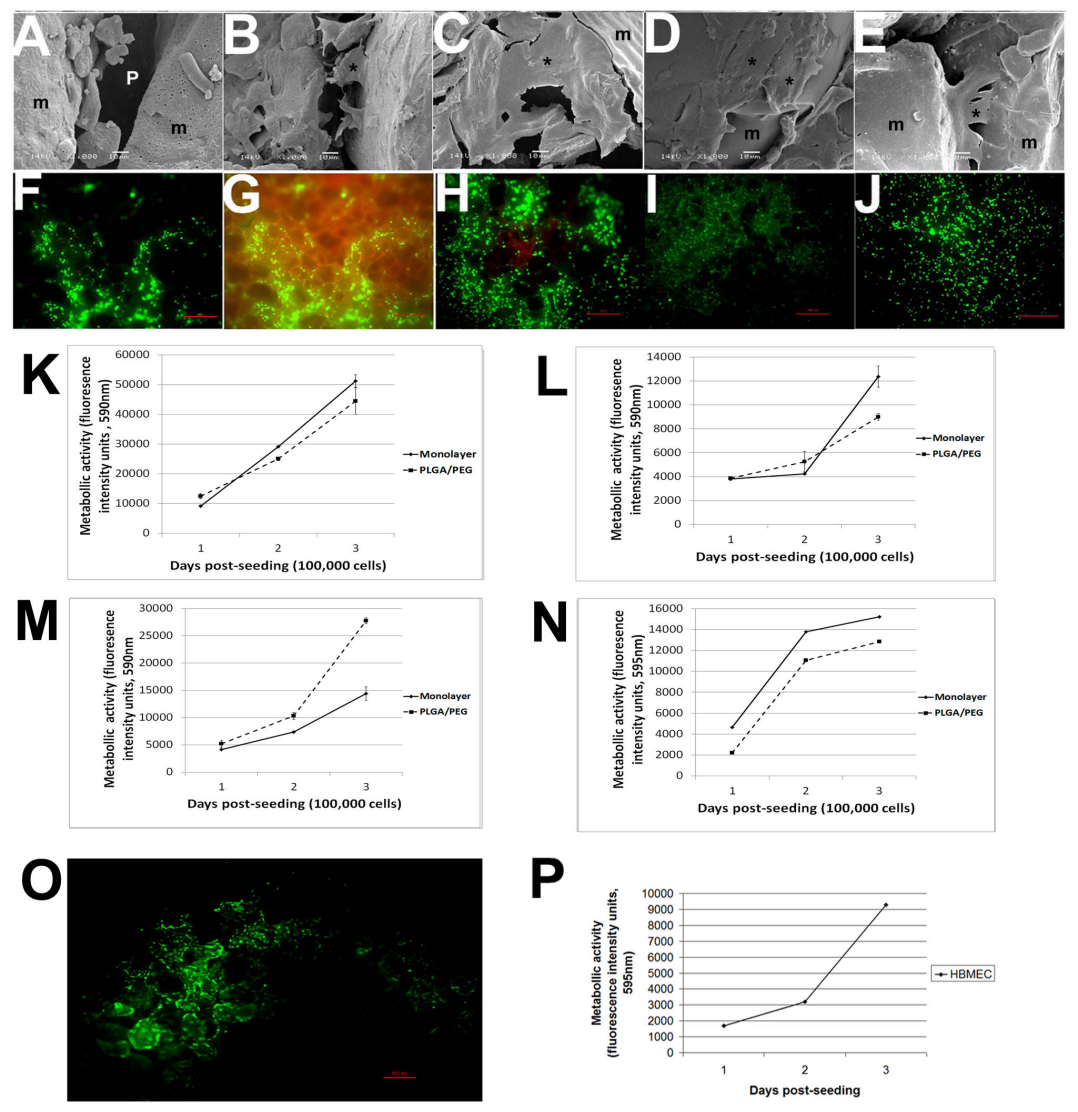
Assessment of PLGA/PEG microparticle-based matrix toxicity to normal and cancerous cells. To address toxicity of polymer matrices, brain cancer cells were grown on PLGA/PEG matrices. (A) Scanning electron microscopy images of blank PLGA/PEG matrices with distinct microparticles (*m*) and pores (*p*). (B-E) PFSK-1 (CNS PNET), DAOY (medulloblastoma), C6 (rat glioma) and U87 (glioblastoma multiforme) brain tumor cells cultured on PLGA/PEG matrices for 3 days. Cells exhibit 3D morphology both across microparticle faces and between microparticles, with extra cellular matrix visible. *Representative tumor cells are denoted with an asterisk*. *Magnifications x1000*. (F) Live/Dead assay of PFSK-1 cells demonstrates that ~85% of cells are viable. (G) Image of PFSK-1 cells without adjustment of background fluorescence, with visible microparticles (background fluorescence from particles is reddish-brown). (H-J) Live/Dead assay of DAOY, C6 and U87 cells demonstrates that 85%-100% of cells are viable. All images are shown on culture day 3 post-seeding onto matrices. *Green = viable; Red = non-viable. Scale bar = 100µm*. (K-N) Alamar Blue proliferation assay for PFSK-1, DAOY, C6 and U87 respectively, over a 3-day culture period post-seeding onto PLGA/PEG matrices and compared to 2D monolayer cultures of the same cell lines. The proportion of proliferating cells was inferred indirectly from a measurement of metabolic activity. Error bars represent the standard error of the mean from three independent matrices and over three independent experiments. Unpaired t-test analyses reveals no significant differences between the two culture systems (O-P) Viability and proliferation of brain endothelial cells (HBMEC) over a 3-day culture period post-seeding onto PLGA/PEG matrices *Green = viable; Red = non-viable. Scale bar = 100µm*.

To address whether PLGA/PEG matrices exert cytotoxicity, PFSK-1, DAOY, C6 and U87 brain tumor cell lines were seeded directly onto matrices and cultured for three days. All seeded tumor cells show distinct 3D morphology both on and between polymer microparticles, with visible extracellular matrix laid on the particle surface (Figures 3a-e). The Live/Dead assay confirmed that ~85% of tumor cells seeded onto matrices remain viable after three days (Figure 3f-j). When regarding a three day culture period, there is no significant difference in metabolic activity of tumor cells grown on matrices compared to the same cell lines cultured as 2D monolayers (PFSK-1 p<0.8, DAOY p<0.4, C6 p<0.2 and U87 p<0.6), thus indirectly demonstrating a comparable proliferation rate over a three day culture period (Figure 3k-n). To verify that the observed lack of cellular toxicity due to PLGA/PEG matrices is not observed exclusively in cancerous cells, normal brain endothelial cells (HBMEC) were seeded onto matrices. Over a three day culture period, endothelial cells were also observed to proliferate with 100% viability (Figure 3o-p). Thus these results demonstrate that PLGA/PEG microparticle-based matrices are non-toxic to neoplastic and normal brain cells.

### PLGA/PEG matrices permit sustained release of chemotherapeutic agents *in vitro*

**Figure 4:**
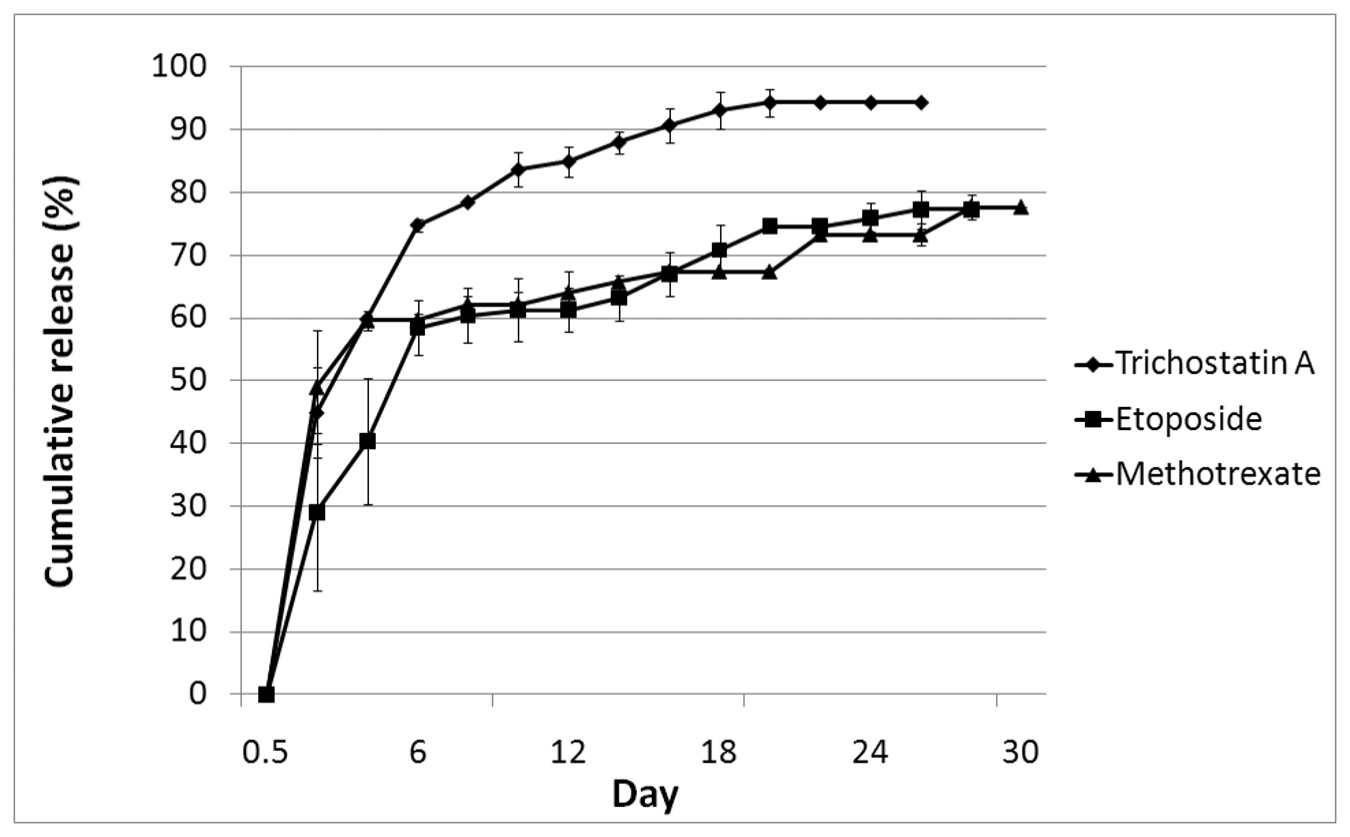
*In* *vitro* cumulative release profiles of agents loaded onto PLGA/PEG matrices. All drug release behaviors from the drug-loaded polymer matrices were investigated under simulated physiological conditions (PBS, pH 7.4) at 37°C. (Top) TSA exhibits an initial burst of 44% of total drug loaded with a total of 94% of drug released by day 18 and no drug detected thereafter. (Middle) ETOP exhibits an initial burst of 29% of total drug loaded with 77% of drug released by day 26. (Bottom) MTX exhibits an initial burst of 49% of total drug loaded with 78% of drug released by day 28. No drug release is detected thereafter. Three independent matrices were used for each experimental set-up with 50µg of drug loaded per matrix. Day 0 initial burst readings were taken 4h after drug-loaded matrices were placed in PBS. UV absorbance wavelengths were as follows: TSA, 342nm; ETOP, 295nm; MTX, 324nm. Error bars represent standard error of the mean from three independent matrices.

To evaluate the capability of PLGA/PEG matrices to release standard of care and experimental chemotherapeutic agents, TSA, ETOP and MTX were loaded onto polymer matrices and drug release profiles measured *in vitro*. Release of TSA, ETOP and MTX followed a similar profile, exhibiting an initial burst phase followed by slower, sustained release profiles (Figure 4a-c). An initial burst release of 44% TSA was observed, caused by drug on the surface of the scaffolds being released into the PBS immediately. This was followed by an average daily release of 1.2% until day 18 (Figure 4, *top*). At this point the PLGA/PEG matrices stopped releasing drug, with a total of 94% TSA released. ETOP-loaded matrices exhibited an initial burst of 29% total drug loaded followed by an average daily release of 1.3% (Figure 4, *middle*). Drug release from the matrices stopped at day 26, with a total of 77% ETOP released. A burst release of 49% MTX was observed followed by an average of 0.7% release per day (Figure 4, *bottom*). The matrices stopped releasing MTX by day 28, at which point 78% total loaded MTX was released.

To determine whether the amount of drug loaded influences the release profile, TSA was loaded onto PLGA/PEG matrices at 50µg and 100µg and drug release profiles directly compared. The initial burst on Day 0 is greater in matrices loaded with 100µg TSA (50%) compared to matrices loaded with 50µg TSA (28%); the release profiles thereafter however, are comparable with a high degree of correlation over an 8-day period (Pearsons correlation coefficient, 0.94). Therefore although the initial burst release of TSA is directly proportional to the amount of drug loaded, the slower sustained zero-order release is independent of the amount of drug loaded (Figure S1).

### Released agents from PLGA/PEG matrices retain cytotoxic capabilities *in vitro*

**Figure 5:**
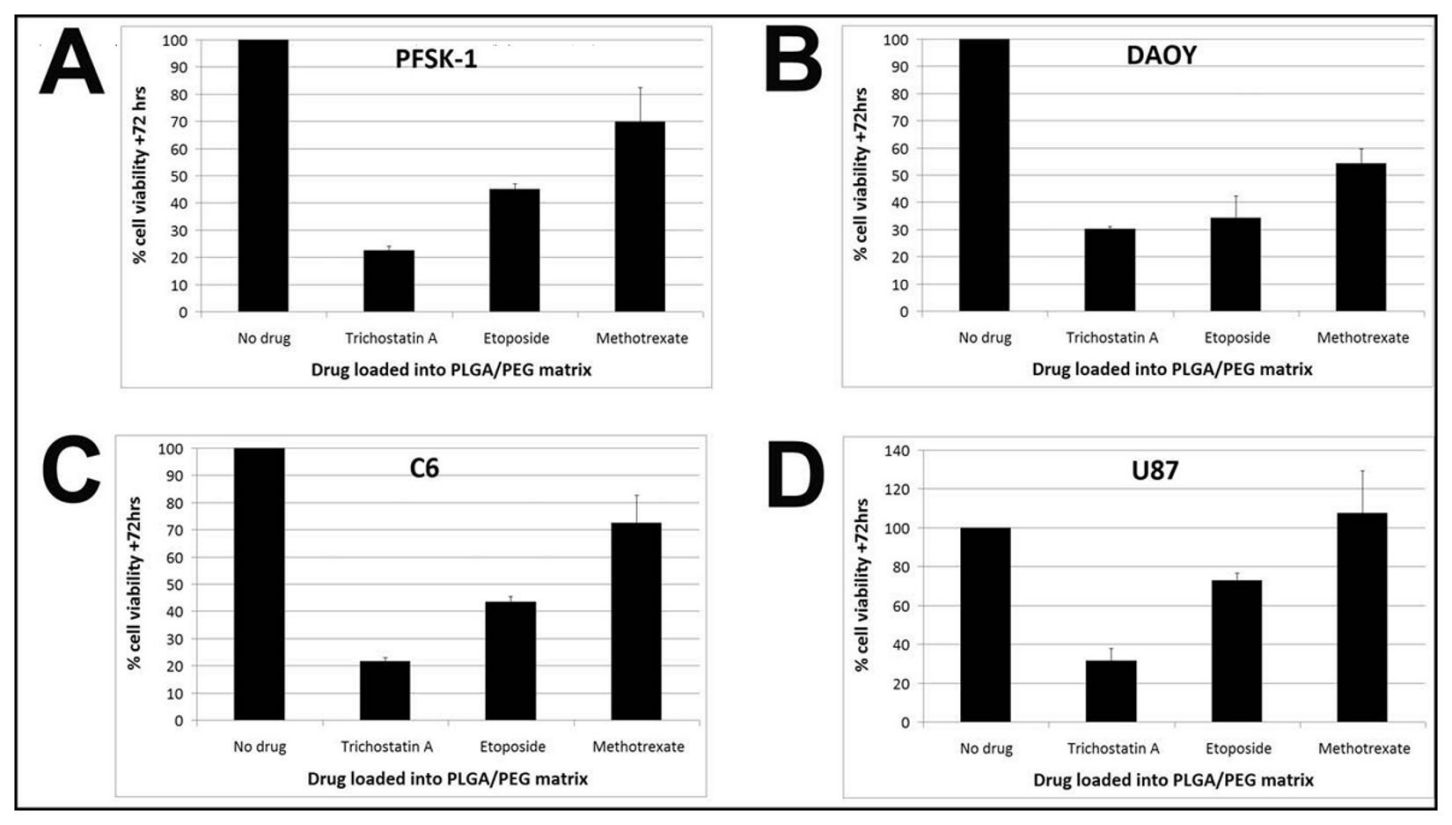
Cytotoxicity of agents released from PLGA/PEG matrices. Brain tumor cells were assessed for viability after seeding onto drug-loaded matrices one day after drugs were loaded, to determine cytotoxic capabilities of released agents. (A-C) PFSK-1, DAOY and C6 cells are sensitive to all three agents with approximately 20-70% viable cells remaining (D) U87 is sensitive to TSA and ETOP with approximately 25-75% viable cells remaining but is insensitive to MTX. *Percentage cell viability was calculated relative to PBS-loaded control matrices for each cell line*.

To determine whether drugs released from PLGA/PEG microparticle-based matrices retain cytotoxic function, brain tumor cells were assessed for viability after seeding onto drug-loaded matrices 24 hours after drugs were incorporated and exposing to released drugs for 72 hours. PFSK-1 is sensitive to all three agents with approximately 20-70% viable cells remaining with drug potency in the order: TSA>ETOP>MTX (Figure 5a). DAOY is sensitive to all three agents with approximately 30-55% viable cells remaining with drug potency in the order: TSA/ETOP>MTX (Figure 5b). C6 is sensitive to all three agents with approximately 20-70% viable cells remaining with drug potency in the order: TSA>ETOP>MTX (Figure 5c). U87 is sensitive to TSA and ETOP with approximately 25-75% viable cells remaining with drug potency in the order: TSA>ETOP. U87 is insensitive to MTX under the experimental conditions described here (Figure 5d). Additionally, TSA cytotoxicity on brain tumor cells was assessed following a 2 week release period *in vitro*, to demonstrate that the released drug retains its activity and is capable of exerting cytotoxic effects after a prolonged period of time within the PLGA/PEG matrix. TSA-loaded PLGA/PEG matrices were placed in PBS for 14 days to allow drug release. On day 14 brain tumor cells were seeded onto matrices and assessed for cell viability after three days of culture. The results indicate that cumulative release of TSA on days 15-17 post drug-loading (~4% of total drug loaded), retains cytotoxic function as cell proliferation is impaired in all four cell lines investigated at IC_50_ concentrations (Figure S2) These results indicate that chemical and physical interaction of TSA, ETOP and MTX with the PLGA/PEG polymer does not impair molecular structure integrity of the drug, as these agents retain cytotoxic function *in vitro*.

### PLGA/PEG matrices release active etoposide *in vivo*

**Figure 6:**
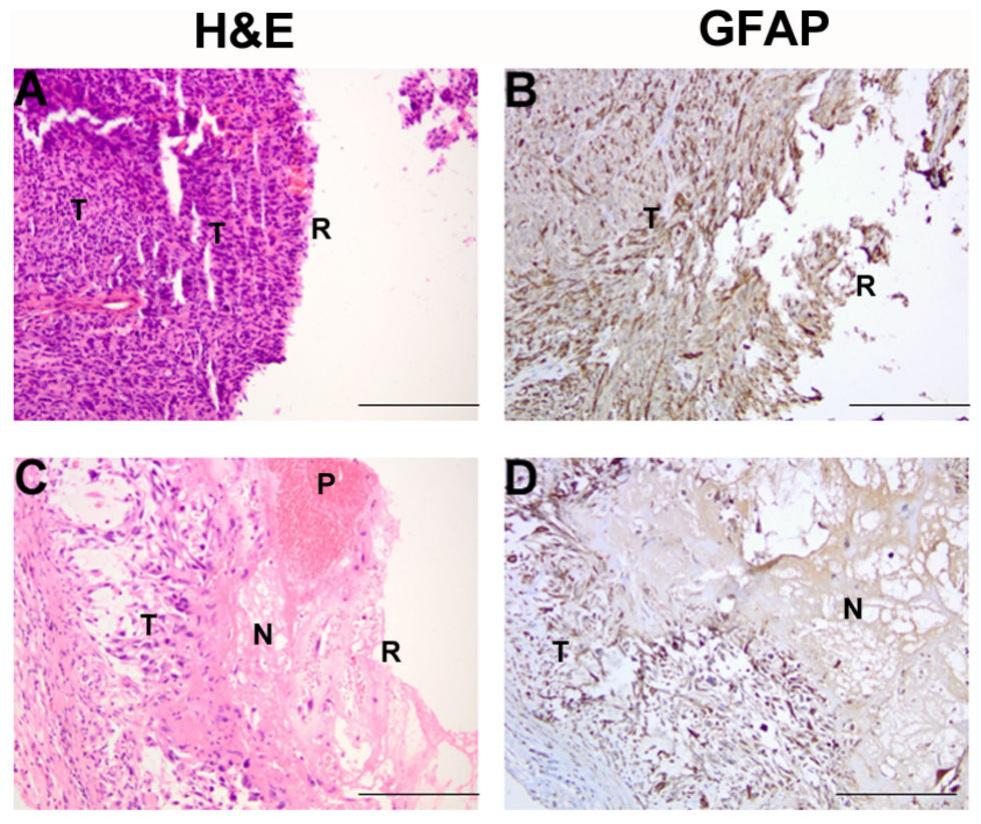
Efficacious release of etoposide-loaded PLGA/PEG in a mouse flank xenograft. Mice were sacrificed 3 days after implantation of either blank (A-B) or etoposide-loaded (C-D) polymer. Haematoxylin and eosin staining shows a clear kill-zone is visible beyond the resection boundary (R) consisting of necrotic tumour cells (N), demonstrating *in* *vivo* release of cytotoxic levels of etoposide from the polymer matrix. A similar result is observed with GFAP staining of tumour xenograft cells. T, viable tumour cells; P, residual polymer implant. *Scale bar = 400µm*.

Etoposide-loaded polymer was moulded around a flank GBM resection cavity after performing partial tumour resection. Mice were sacrificed 3 days after implantation of the drug-loaded polymer to demonstrate proof-of-concept for *in vivo* drug release and gauge short-term drug diffusion distance. Haematoxylin and eosin staining shows a clear kill-zone visible beyond the resection boundary consisting of necrotic tumour cells, demonstrating *in vivo* release of cytotoxic levels of etoposide from the polymer matrix. Etoposide drug diffusion after release from the polymer matrices was approximately 1mm after 3 days, based indirectly upon the region of the kill-zone (Figure 6).

## Discussion

The BBB acts as a block to the administration of many chemotherapeutic agents to the central nervous system. Drugs have to be given at escalating doses to achieve effective doses at the tumor site, but this increased dosing leads to significant side-effects and toxicities systemically, e.g. bone marrow suppression, nausea or epithelial damage. By administering chemotherapy directly into the brain tumor cavity, thus bypassing the BBB, high local doses can be achieved to ensure maximal anti-cancer activity, whilst minimizing systemic toxicity. The proof of concept of such an approach has been achieved by Gliadel®; however despite its potential, treatment with Gliadel® has been variable in efficacy due to drug resistance in patients [36] and the technical product characteristics of Gliadel®. The rate of release from Gliadel® is also essentially uncontrolled with the majority of drug release occurring within a few days of implantation, reducing the chronicity of effect (both *in vitro* release in saline buffer at 37°C and *in vivo* studies document a release period of 5 days) and compounding its limited success in the clinic [25]. 

Here we document a PLGA/PEG microparticle-based matrix that provides a novel application mode of a biomaterial in close proximity to the tumor bed and micro-deposits of neoplastic cells directly beyond. When mixed with saline, the PLGA/PEG microparticle formulation is a paste onto which chemotherapeutics can be loaded. This paste is easily molded around a tumor resection cavity after surgery and begins to solidify into a matrix at body temperature, adhering to the surgical cavity wall. Close tissue apposition of the PLGA/PEG matrix to the cavity wall as observed when applied surgically *ex vivo*, refines and maximizes the potential of this therapeutic modality. The ability to mould the local chemotherapy source around the entire irregular shaped resection cavity offers vastly superior tissue approximation when compared to discrete polymeric wafers or polymer-based hydrogels. To our knowledge, this is the first report of polymer-based drug delivery system which can be applied as a paste directly onto the brain surface and molded to the shape of that topography.

It is imperative that the clinical utility of the PLGA/PEG matrix be evaluated at this proof-of-concept stage prior to advancement to *in vivo* studies and early phase patient trials. Our findings indicate that the PLGA/PEG polymer does not interfere with MRI and CT scanning of the brain using standard clinical sequences. No polymer-related artifact was evident in either scan modality, with biomaterial clearly distinguishable from brain parenchyma. Therefore it is unlikely that the presence of the polymer lining a resected cavity would impair visualization of tumor recurrence in a patient following MRI or CT scanning. Furthermore, a typical high dose fractionated radiotherapy course delivered to a patient with intra-cavity PLGA/PEG matrices, is unlikely to affect the sustained and gradual release of chemotherapy from the polymer, as no alteration in the microstructure of the matrix was observed using an anthropomorphic ‘dummy’ phantom. Specifically, the porosity of the microparticles did not appear to differ from non-irradiated polymer, implying that high dose radiation is unlikely to cause the remaining drug to be released in a rapid burst due to the loss of structural integrity of the polymer.

Exposing brain tumor cells and brain endothelial cells to PLGA/PEG matrices by seeding cells directly onto matrices allows an indication of whether the polymer formulation is toxic. Our findings comprehensively show that both neoplastic and untransformed cells are viable and proliferate during at least a 3-day culture period. This is consistent with the documented low cytotoxicity and good biocompatibility of resorbable FDA-approved PLGA medical sutures [31,32]. Similarly, a recent study using rats with C6 glioma cells orthotopically implanted, observed no significant difference in survival time between blank PLGA microparticles interstitially delivered and untreated control animals, suggesting that PLGA had no inhibitory effect on C6 cells [37]. Our results suggest that our specific formulation of PLGA/PEG does not result in cellular toxicity; however biocompatibility assessment of the PLGA/PEG matrix with host tissue using *in vivo* models and during patient trials will be required to validate these *in vitro* findings.

Pre-clinical polymer-based drug delivery systems targeting localized brain tumor chemotherapy have predominantly utilized single-agent drug release strategies or those involving only one class of drug (e.g. hydrophobic, lipophilic etc.) [12,15,38–41]. Many of these studies, including those investigating the *in vivo* pharmacokinetics of the carmustine implant (Gliadel®), have achieved only up to seven days drug release at which point carmustine release was almost complete [25,42]. We have demonstrated 18-28 days sustained drug release using two standard of care chemotherapeutic agents and one experimental anti-cancer agent shown by us previously to exert anti-tumor effects on childhood brain tumor cells *in vitro* [43]. These drugs are grouped into two distinct drug classes: TSA, lipophilic; ETOP and MTX, hydrophobic. Release profiles of TSA, ETOP and MTX from PLGA/PEG matrices generally follow a biphasic profile, at least for the duration of the described experiments [44]. Our principal outcome measure in this objective was to achieve 3-4 weeks drug release as this typically represents the lag period between surgical resection and commencement of standard chemotherapy administration. Therefore this would potentially permit oncological (local) treatment as an adjuvant therapy prior to standard systemic administration. A characteristic initial burst of between ~30-50% is released rapidly and corresponds to drug molecules bound to the periphery of the microparticle-based matrix and attached to the surface of the microparticles. A sustained release phase follows where 0.7-1.3% of drug is released per day and corresponds to drug trapped within the pores between microparticles. Release in this second scenario occurs via diffusion through the pores. It is likely that an additional phase would occur upon full degradation of the PLGA/PEG polymer, at which point the remainder of the drug would be anticipated to be released. Although we observed a cessation of MTX release from day 4 to 7, drug release thereafter continued again from day 8 and continued at a low level until ~80% of the loaded drug was released from the matrices. This cessation of MTX release is consistent with a recent report using chitosan-based nanoparticles for MTX delivery to brain tumors, which documents a 3-day release profile with ~80% of MTX still entrapped within the nanoparticles [41]. In contrast, almost all of TSA is released from PLGA/PEG matrices *in vitro*. Our result shows sustained release of TSA over 18 days, whereas a recent study using liposomes loaded with TSA reports 100% drug release over 24 hours [45]. Similarly, our results show a more sustained release profile of ETOP over 24 days, compared to a recent report using poly(ether-anhydride) particles, which releases ETOP *in vitro* over 6 days [40]. The *in vitro* drug release assay described in this study provides a screening method for assessing drug suitability with this delivery system. Importantly, the physicochemical interactions between these chemotherapeutics and PLGA do not impair the drug activity as demonstrated by the capability of drugs released from PLGA/PEG matrices to exert a cytotoxic effect on brain tumor cells. Moreover, short-term *in vivo* studies demonstrate proof-of-concept for drug release within a tumor tissue microenvironment and approximately 1mm drug diffusion distance. Longer-term *in vivo* studies will be required to corroborate this finding and to determine maximal drug diffusion distance.

In summary, we describe a novel intra-cavity drug delivery system using a PLGA/PEG microparticle-based matrix, which can be easily pasted around the resected tumor bed. Multiple chemotherapeutic agents can be loaded onto this polymer which solidifies at body temperature, gradually releasing the drugs over time. The PLGA/PEG matrix is amenable to use with current standard clinical procedures as polymer can be distinguished during MRI/CT scanning and radiotherapy dosing does not adversely affect the polymer structure. The polymer is non-toxic to tumor and normal cells and the chemotherapeutic agents TSA, ETOP and MTX can be released from matrices in a sustained manner over 3-4 weeks *in vitro* with retention of cytotoxic capability. Our results should expedite *in vivo* studies of efficacy and neurotoxicity using combinations of standard of care agents and ultimately lead to early phase patient trials for the treatment of high grade brain tumors. Although described here for the treatment of childhood and adult brain tumors, this system has applicability to any solid tumor for which complete surgical resection of the tumor is not achievable.

## Supporting Information

Figure S1 **TSA release profiles at different amounts of drug loading.** Drug loading at both 50µg and 100µg show similar sustained release profiles over an experimental period of 8 days, after a concentration-dependent initial release burst (28% vs. 50% respectively).
(TIF)

Figure S2 **Cytotoxicity of TSA released *invitro* between days 15-17.** Brain tumor cells were seeded onto TSA-loaded PLGA/PEG microparticle-based matrices 14 days after drugs were loaded and assessed for viability after 72h. Cumulative TSA release between days 15-17 post drug-loading retains cytotoxic capability as proliferation is impaired in PFSK-1, DAOY, C6 and U87 brain tumor cells. Brain tumor cells seeded onto PBS-loaded matrices were used as controls.
(TIF)

Movie S1 **Application of PLGA/PEG microparticle-based paste onto pseudo-resection wall.** When mixed with a saline carrier solution, PLGA/PEG microparticles form a paste that can be easily molded around an ovine pseudo-resection cavity *ex* *vivo*.
(MPG)

Movie S2 **PLGA/PEG matrix post-sintering *exvivo*.** Within 15 minutes at 37°C, particles solidify to form a matrix that retains close apposition to the resection cavity wall.
(MPG)
