# Supplementary figures and images for: Adjuvant Chemotherapy for Brain Tumors Delivered via a Novel Intra-Cavity Moldable Polymer Matrix
Source: PLoS One. 2013 Oct 14;8(10):e77435. doi: 10.1371/journal.pone.0077435 (PMC3796488; doi:10.1371/journal.pone.0077435)

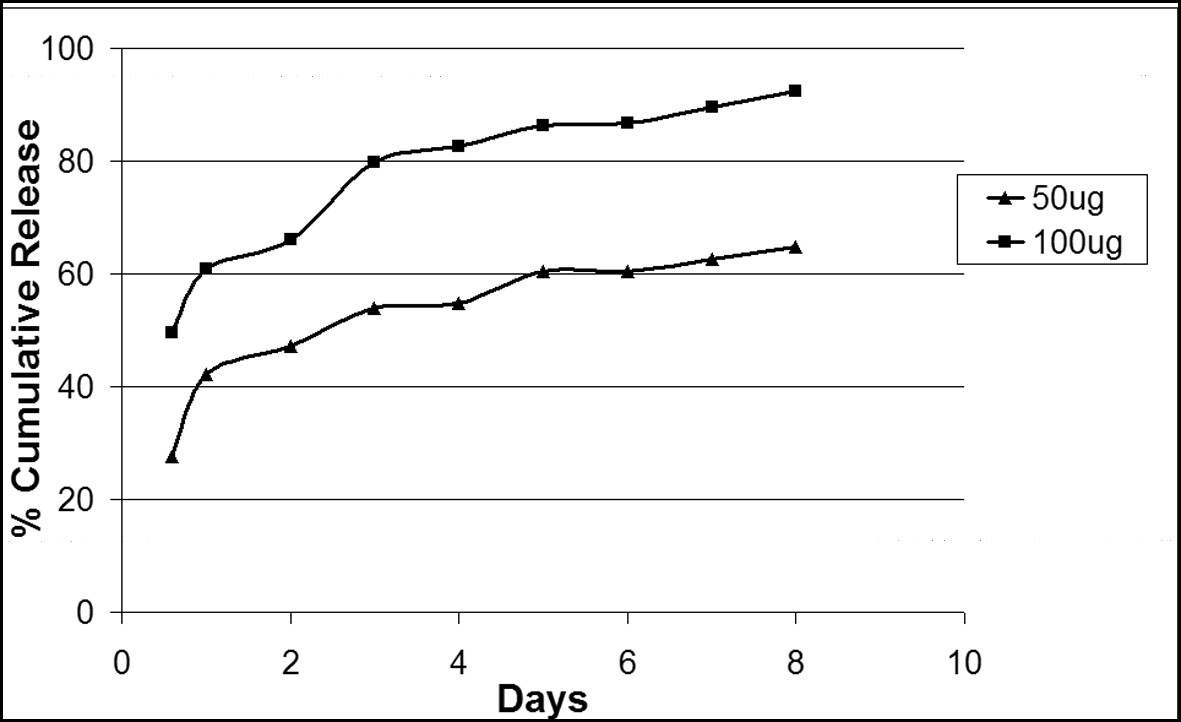

Supplement: Figure S1 — TSA release profiles at different amounts of drug loading. Drug loading at both 50µg and 100µg show similar sustained release profiles over an experimental period of 8 days, after a concentration-dependent initial release burst (28% vs. 50% respectively). (TIF) [file pone.0077435.s001.tif]

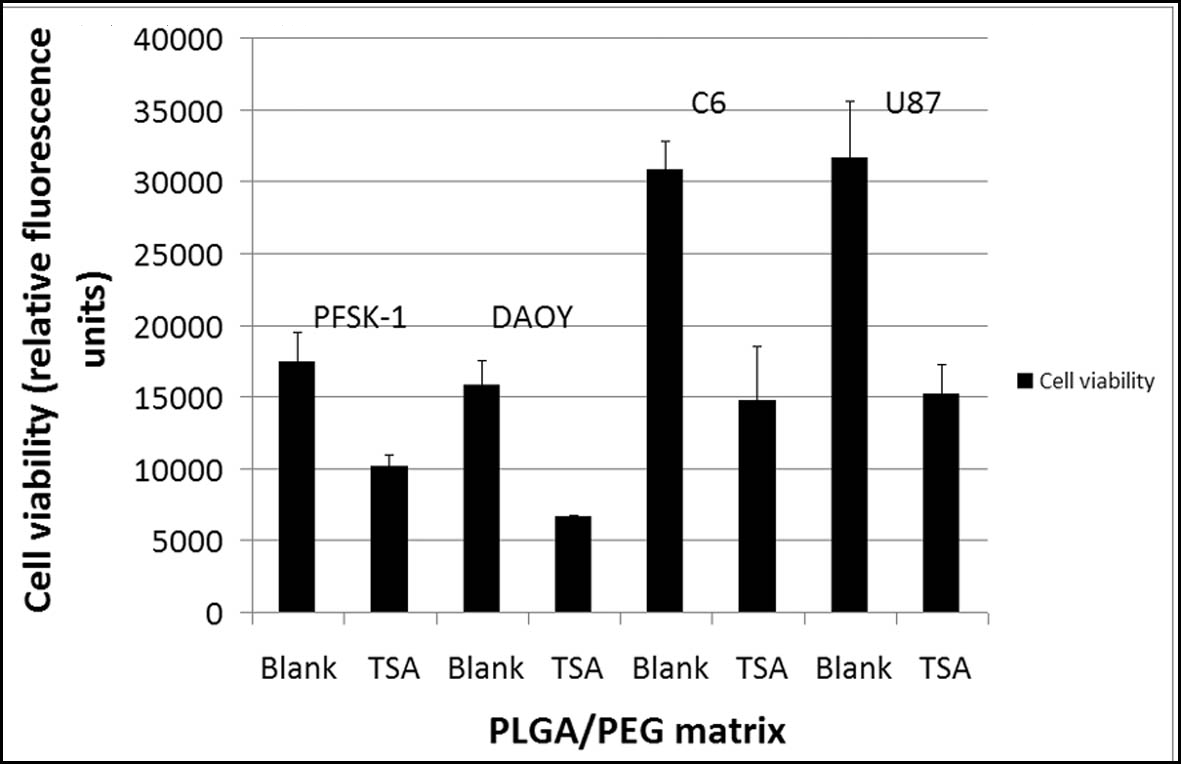

Supplement: Figure S2 — Cytotoxicity of TSA released invitro between days 15-17. Brain tumor cells were seeded onto TSA-loaded PLGA/PEG microparticle-based matrices 14 days after drugs were loaded and assessed for viability after 72h. Cumulative TSA release between days 15-17 post drug-loading retains cytotoxic capability as proliferation is impaired in PFSK-1, DAOY, C6 and U87 brain tumor cells. Brain tumor cells seeded onto PBS-loaded matrices were used as controls. (TIF) [file pone.0077435.s002.tif]
